# Electrochemical quantification of d-glucose during the production of bioethanol from thermo-mechanically pre-treated wheat straw

**DOI:** 10.1016/j.elecom.2021.106942

**Published:** 2021-03

**Authors:** Rhys A. Ward, Adam Charlton, Kevin J. Welham, Paul Baker, Sharif H. Zein, Jeremy Tomkinson, David I. Richards, Stephen M. Kelly, Nathan S. Lawrence, Jay D. Wadhawan

**Affiliations:** a Department of Chemical Engineering, The University of Hull, Cottingham Road, Kingston-upon-Hull HU6 7RX, United Kingdom; b Aura Innovation Centre, Bridgehead Business Park, Meadow Road, Hessle HU13 0GD, United Kingdom; c BioComposites Centre, Bangor University, Alun Roberts Building, Bangor, Gwynedd LL57 2UW, Wales, United Kingdom; d Department of Chemistry and Biochemistry, The University of Hull, Cottingham Road, Kingston-upon-Hull HU6 7RX, United Kingdom; e National Non-Foods Crops Centre (NNFCC), Ltd., Biocentre, York Science Park, Innovation Way, York YO10 5DG, United Kingdom

**Keywords:** Bioethanol, Lignocellulosic biomass, Pre-treatment, Wheat straw, Thermo-mechanical refining, Glucose oxidase, Mechanochemistry

## Abstract

Mechanical pre-treatment (disc refining) of wheat straw, at both atmospheric and elevated pressure, is shown to be an efficient process to access fermentable monosaccharides, with the potential to integrate within the infrastructure of existing first-generation bioethanol plants. The mild, enzymatic degradation of this sustainable lignocellulosic biomass affords *ca.* 0.10–0.13 g/g (dry weight) of d-glucose quantifiable voltammetrically in real time, over a two hundred-fold range in experimental laboratory scales (25 mL to 5.0 L), with pressure disc refining of the wheat straw enabling almost twice the amount of d-glucose to be generated during the hydrolysis stage than experiments using atmospheric refining (0.06–0.09 g/g dry weight). Fermentation of the resulting hydrolysate affords 0.08–0.10 g/g (dry weight) of ethanol over similar scales, with ethanol productivity at *ca.* 37 mg/(L h). These results demonstrate that minimal cellulose decomposition occurs during pressure refining of wheat straw, in contrast to hemicellulose, and suggest that the development of green, mechanochemical processes for the scalable and cost-effective manufacture of second-generation bioethanol requires improved cellulose decomposition.

## Highlights

• In-line electroanalysis of d-glucose during bioethanol production.
• Second-generation biosensor suitable for industrial process analytical technology.
• Greater d-glucose yield from wheat straw refined under pressure.
• d-Glucose productivity is 20–40 mg/(L h) over a 5 L scale.

## Graphical abstract

**Figure:**
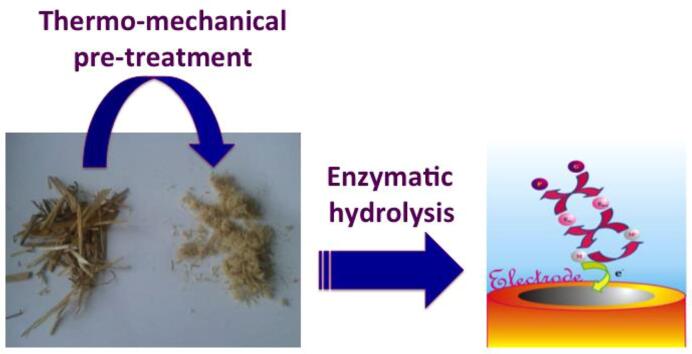

## 1 Introduction

Ethanol is an important bulk chemical [1], [2], used as a fuel/fuel additive, industrial solvent and disinfectant, *cf*., disinfectants for Covid-19. Although ethanol is primarily manufactured through the hydration of ethylene, bioethanol, which is produced from fermentation of sugars in materials obtained from recently harvested plants, has been becoming increasingly more important as part of international strategies to reduce greenhouse gas emissions, thereby assisting in the abatement of climate change [3]. Accordingly, the use of lignocellulosic biomass, such as straw, has been encouraged for “second-generation” bioethanol production. The use of agricultural residues as alternative feedstocks, which are non-edible by humans, also contributes to the legislative targets in regions like the EU, which require reductions in the volumes of first-generation, crop-based biofuels that are currently produced [4]. Moreover, wheat straw is a by-product from wheat, and as such, is an *economically elastic commodity* – its supply moves with the changing demand for the primary food source based on local demographic need [5]: over the last 60 years in the United Kingdom, the production of wheat has tripled [6], [7], with yields ranging between 3.9 and 8.5 tonnes/ha (wheat yields are dependent on the type of soil, climate and the variety of wheat sown); the derived straw yield is typically between 2 and 5 tonnes/ha [8], [9], [10], so that 0.24–1.3 tonnes of straw are produced per tonne of wheat. Thus, benchmarking the first-generation ethanol-to-dry feed ratio as 0.36–0.44 L/kg (0.28 – 0.35 g/g dry weight at 20 °C; this figure actually corresponds to maize not wheat [2]) and noting that, over the next decade, *global* ethanol production for fuel is anticipated to increase at a rate of 1 billion L *p.a.*, to 131 billion L [11], at least 325 Mt of feedstock will be required from *ca.* 55 Mha *per annum* – equivalent to approximately ten times the amount of arable land currently available in the United Kingdom [12], and which emphasises the need to develop efficient processes for second-generation biofuels; second-generation feedstocks afford comparable bioethanol yields (0.2–0.5 g/g) [13], [14], [15], [16]. Note that whilst we prefer to consider g/g dry weight as the most appropriate formulation for yields from biomass used, the literature often corrects for the fraction of cellulose within the biomass considered [17].

**Fig. 1:**
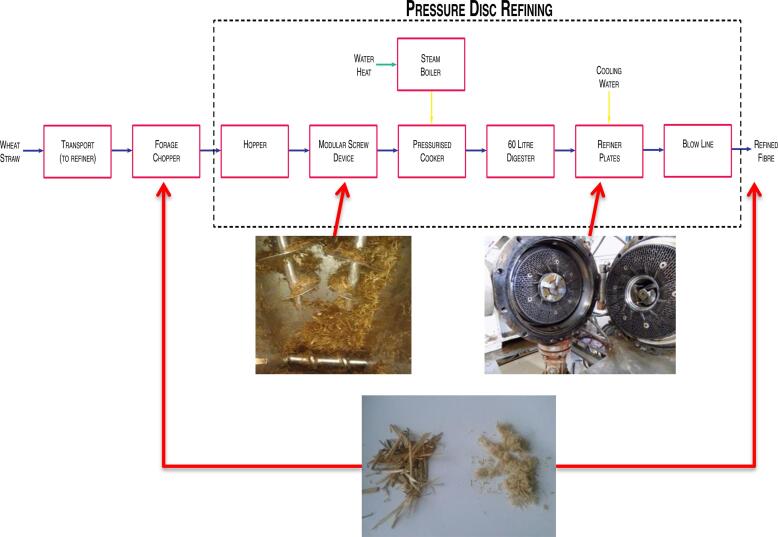
Thermo-mechanical pre-treatment process used for the production of refined wheat straw fibre (top), with the modular screw device (MSD) and refiner plates showing the configuration of bars and grooves illustrated (middle). The difference in particle size between forage chopped wheat straw fibre and pressure-disc refined material is depicted (bottom).

Lignocellulosic biomass typically comprises 33% cellulose, 28% hemicellulose, 24% lignin and 15% of other materials interconnected within a fibrous network [18], [19], [20], which need to be broken down, through pre-treatment and downstream enzymatic hydrolysis [21], to liberate fermentable sugars. In recent work [22], it was demonstrated that a simple and effective pre-treatment of wheat straw under mild conditions, *viz.*, 60 s steam treatment under 6–10 bar and 140–180 °C followed by mechanical disc refining of wheat straw under 6–10 bar, *q.v.* Fig. 1, rendered the cellulose more accessible to subsequent enzymatic hydrolysis at laboratory scale (25 mL). For this process to be viable for bioethanol manufacture, it needs to be scalable, and this requires an evaluation of the conversion efficiency of thermo-mechanically pre-treated wheat straw to bioethanol compared to that achieved using untreated material. A key aspect of this approach is the determination of the amount of d-glucose liberated during the enzymatic hydrolysis, since this level is a convenient index of the “fermentability” of a particular hydrolysate broth, since all yeasts have hexakinases, which metabolise this molecule [23]. In this article, we propose the real-time monitoring of d-glucose levels through voltammetric measurements, using a second-generation biosensor, and demonstrate the potential utility of electrochemical methods in process analytical technologies.

## 2 Results and discussion

The composition of the wheat straw fibres used in this work (see Supplementary Information, SI1 for experimental conditions) is given in Table 1. Compared with atmospherically refined (AR) wheat straw, whilst there is no significant change in the cellulose content as a result of pressurised refining (PR), the hemicellulose content decreases with increasing pressure used in the thermo-mechanical process; in contrast, the non-fibrous content increases with refining pressure. The decrease in hemicellulose content is attributed to its decomposition to smaller, soluble sugars, which are responsible for the increase in the non-fibrous content [22].

**Table 1:** Experimental parameters used for the thermo-mechanical refining of wheat straw used in this work, together with compositional analysis of the fibres.

Sample Code and Composition	Pre-treatment conditions	Refiner plate gap/μm	Length of refining time at each pressure/min
Atmospheric refining (AR) Composition:†, ‡ Non-fibre 24.8% Hemicellulose 31.8% Cellulose 36.0% Lignin 5.4% Theoretical monosaccharide concentration:† 0.75 g/g dry biomass	Forage chopper (0.5 in) followed by atmospheric disc refining (AR) 2% Consistency Single pass, using re-sharpened refiner plates	0	N/A
Pressurised disc refining at 6 bar (PR6) Composition:†, ‡ Non-fibre 25.8% Hemicellulose 28.1% Cellulose 32.8% Lignin 8.8% Theoretical monosaccharide concentration:† 0.65 g/g dry biomass	Forage chopper (0.5 in) followed by pressure disc refining (PR) at 6 bar, , using un-sharpened ‘low intensity’ Andritz refiner plates (type D2-516)	15	20
Pressurised disc refining at 8 bar (PR8) Composition:‡ Non-fibre 28.2% Hemicellulose 21.8% Cellulose 29.8% Lignin 13.4% Theoretical monosaccharide concentration:† 0.6 g/g dry biomass	Forage chopper (0.5 in) followed by pressure disc refining (PR) at 8 bar, using re-sharpened ‘low intensity’ Andritz refiner plates (type D2-516)	4	15
Pressurised disc refining at 10 bar (PR10) Composition:‡ Non-fibre 36.1% Hemicellulose 15.0% Cellulose 29.3% Lignin 12.2% Theoretical monosaccharide concentration:† 0.7 g/g dry biomass	Forage chopper (0.5 in) followed by Pressure disc refining (PR) at 10 bar, using new ‘high intensity’ Andritz refiner plates (type D2-503)	4	15

† Estimated from data provided in reference [22].

‡ The remainder is ash.

**Fig. 2:**
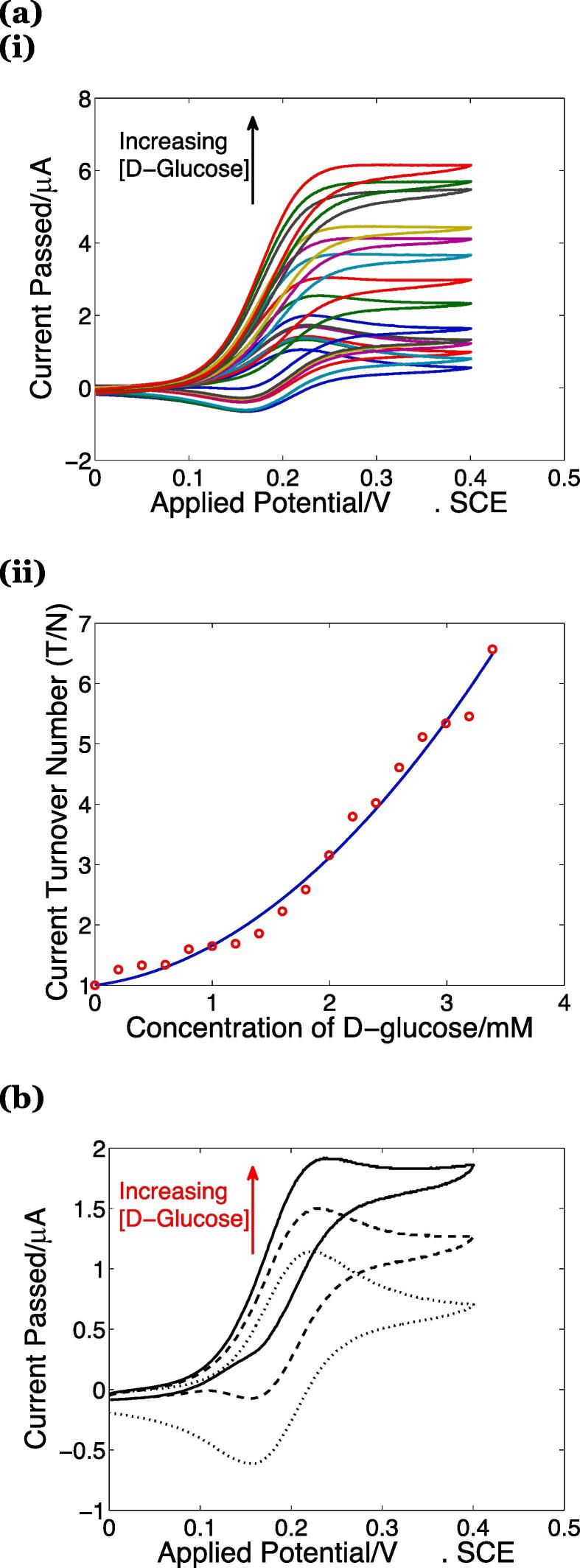
(a) (i) Cyclic voltammograms (0.1 V/s) at a 3 mm diameter glassy carbon electrode of 0.26 mM ferrocenemethanol in 0.1 M aqueous phosphate buffer at pH 6.74, 20 ± 2 °C containing 0.1 mg/mL GOx, with and without 1.0 μL standard additions of a 1.0 M aqueous glucose solution (mutarotated). The arrow indicates increasing d-glucose concentration in the solution. (ii) Calibration curve expressed in terms of total d-glucose added, created through analysing the current peak and plateau data from (i) using equation (S1, S2), red circles. The solid blue line represents the line of best-fit used for analysing the hydrolysate data in panel (c), with equation $T/N=0.4031{\left(c_{G}^{0}\right)}^{2}+0.2540c_{G}^{0}+1.000$ and coefficient of determination R^2^ = 0.9865, and where c_G_^0^ is the bulk d-glucose concentration expressed in mM.(b) Cyclic voltammograms (0.1 V/s) at a 3 mm diameter glassy carbon electrode of 0.26 mM ferrocenemethanol in 0.1 M aqueous phosphate buffer at pH 6.74, 20 ± 2 °C containing 0.1 mg/mL GOx, after 0 μL (dotted), 100 μL (dashed) and 200 μL (solid) additions of filtered hydrolysate sample derived from 10 bar pressure-refined wheat straw hydrolysed at an experimentation scales of 25 mL. The arrow indicates increasing d-glucose concentration in the solution. (For interpretation of the references to colour in this figure legend, the reader is referred to the web version of this article.)

**Fig. 3:**
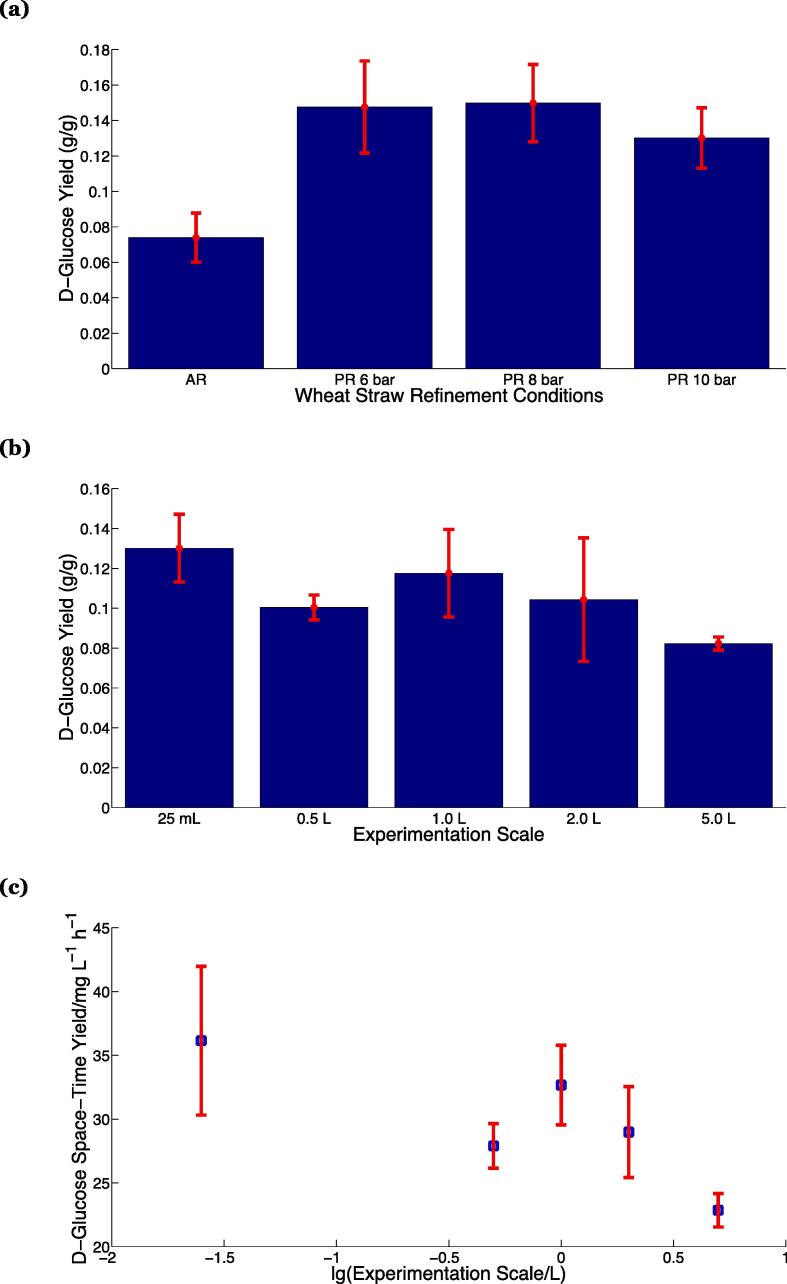
(a) Mass-by-mass d-glucose (dry weight) yields corresponding to an experimental scale of 25 mL for atmospheric disc refined (AR, n = 12), 6 bar pressure refined (PR6 bar, n = 12), 8 bar pressure refined (PR8 bar, n = 18) and 10 bar pressure refined (PR10 bar, n = 24) wheat straw.(b) Mass-by-mass d-glucose yields (dry weight) corresponding to the hydrolysis of 10 bar pressure refined wheat straw at different experiment scales.(c) Productivity of d-glucose through enzymatic hydrolysis of PR10 fibres with experimental scale. In (b) and (c), the experimental scale correspond to 25 mL (n = 24), 0.5 L (n = 4), 1.0 L (n = 14), 2.0 L (n = 10), 5.0 L (n = 6); in (a), (b) and (c), n represents the sample size; the red error bars represent one standard deviation. (For interpretation of the references to colour in this figure legend, the reader is referred to the web version of this article.)

In order to monitor the extent of d-glucose formation through enzymatic hydrolysis of the wheat straw fibres, protocols, developed as described in SI2, were used. These experiments rely on the electrochemical mediation of glucose-1-oxidase by soluble one-electron homogeneous mediators, such as ferricenium ions, in a manner used in commercial blood glucose sensors for the management of diabetes [24]. The advantages of this second-generation biosensor approach are identified in SI2, and include the specificity to β-d-glucose (even over the α-anomer), with at least two-orders of magnitude greater activity towards d-glucose over other sugars derived from hemicellusose (d-xylose, d-mannose, d-galactose) [25], [26], with D–rhamnose identified as not being oxidised [25] and D–arabinose acting as an inhibitor [26]. Thus, even if hemicellulose is broken down, the voltammetric measurement kinetically discriminates between the sugars. Moreover, advantageously, this approach enables a real-time measurement of d-glucose, and thus may require less dilution compared with HPLC methods. For quantitative determination of β-d-glucose in hydrolysate, voltammograms of ferrocenemethanol (mediator) were recorded at 0.1 V/s at a glassy carbon electrode in the presence of glucose-1-oxidase and various concentrations of β-d-glucose, see Fig. 2a(i), so as to produce the external calibration curve shown in Fig. 2a(ii). This graph shows the total d-glucose concentration as both glucose anomers are metabolised by yeast cells. Thus, voltammograms recorded by the addition of hydrolysate (*q.v.* Fig. 2b) were analysed in terms of the current turnover number (T/N). The observed maximum current in the presence of β-d-glucose, expressed relative to that in its absence, affords the results presented in Fig. 3 for the experimental matrix considered.

At a constant experiment scale (25 mL), hydrolysate d-glucose concentrations were found to range between *ca.* 14–17 mM for all of the PR samples considered, essentially twice that observed from the AR samples (*ca.* 8 mM). The latter yield corresponds to between 60 and 90 g/kg dry biomass, and compared favourably with that observed in the literature from milled wheat straw (54 g/kg dry biomass) [27]. These results imply a more sugar-rich feed-base for the PR samples; it is evident from Fig. 3a, that the liberated mass of d-glucose per dry biomass is essentially constant for the PR samples and significantly larger than that derived from the AR samples. This result is in line with previous work, which investigated the level of total monosaccharides after enzymatic hydrolysis [22]. Since the cellulose level is approximately constant in the samples, but the hemicellulose content decreases with refining pressure, these results appear to indicate that a large proportion of the degradation products converting into sugars are derived from hemicellulose, either through direct decomposition during pressurised disc refining or enzymatic hydrolysis. The reason underpinning this is likely to be the combination of steam and disc refining, which softens and then disrupts the biomass matrix, thereby exposing the cellulose fraction and enabling easier access for the enzyme through reduced recalcitrance [22]. Note that the slightly smaller amounts of d-glucose liberated from the PR10 samples compared with the PR8 or PR6 samples is likely due to plate effects – the former were refined using 503 plates; the latter with 516 plates. This is in agreement with previously reported results for the release of total monosaccharides, being *ca.* 0.3 g/g dry biomass for PR10 refined at 503 plates *vs.* 0.5 g/g dry biomass for PR8 samples refined at 516 plates [22].

In order to investigate the scalability of the process, hydrolyses were undertaken using 10 bar PR samples at volumes differing by over two orders of magnitude (25 mL to 5.0 L). The results, reported in Fig. 3b, correspond to 12–14 mM d-glucose concentration over a range of 25 mL to 2.0 L, with a slightly lower concentration (*ca.* 9 mM) for the largest volume experiments (5.0 L). Consolidation of these data with the experimental methods used for the hydrolysis reveals that under a constant rate of mixing, there is very little statistical difference in the concentration of d-glucose liberated with experimental scale, as expected. The data pertaining to the 5.0 L scale experiments are slightly exceptional – they correspond to an altered mixing regime as discussed in SI3. This interpretation is reinforced through the scale-variation of the productivity, illustrated in Fig. 3c. The space–time yield [28] slightly decreases with experimental scale from 36.1 ± 5.8 mg/(L h) at the smallest experimental scale (25 mL), dropping by over 35% to 22.9 ± 1.3 mg/(L h) at the largest scale (5 L).

Nevertheless, over a two hundred-fold volumetric scale ranging 4.975 L, our protocols yield between 0.10 and 0.13 g/g dry weight d-glucose under a wide scale of experimentation volumes, which are in accordance with previously reported [29], [30] literature values (0.19–0.43 g/g).

**Fig. 4:**
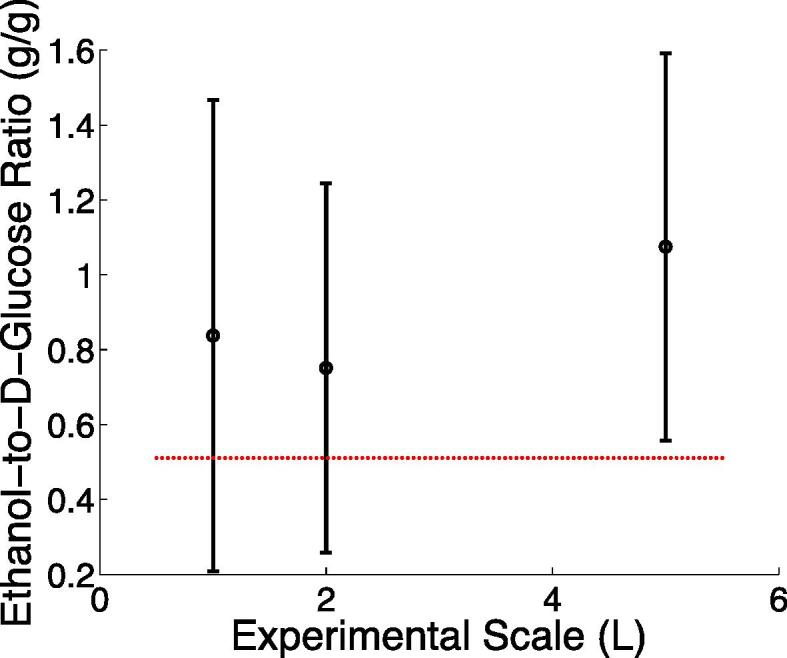
Variation of the ratio of ethanol-to-d-glucose produced from hydrolysis and subsequent fermentation of PR 10 samples. The number of samples studied (n) were 4 (1.0 L), 5 (2.0 L) and 3 (5.0 L), each analysed in triplicate. The error bars indicate one standard deviation. The red line corresponds to the stoichiometrically weighted ratio of the molar masses of ethanol-to-d-glucose. (For interpretation of the references to colour in this figure legend, the reader is referred to the web version of this article.)

Ethanol produced through the fermentation of d-glucose containing hydrolysates with *Saccharomyces cerevisæ* (*q.v.* SI1), was analysed according to the protocols given in SI4, with quantification undertaken by GC–MS with an internal standard (*iso*-propyl alcohol). The results reported in Fig. 4 indicate that ethanol is produced from the fermentation process at 0.22 ± 0.12 vol%, corresponding to *ca.* 0.08–0.10 g/g (dry weight), giving rise to a space–time yield of 37.1 ± 22.9 37 mg/(L h). As indicated in Fig. 4, these are approximately in agreement with the stoichiometric amount of ethanol that can be produced from the amount of d-glucose present (0.51 g/g), although the errors are large, likely as a result of subsampling, given that wheat straw is an highly heterogeneous material.

## 3 Conclusions

In summary, over a two hundred-fold experimental scale, d-glucose at 0.10–0.13 g/g dry weight can be obtained from PR wheat straw, as determined using a second-generation electrochemical biosensor, yielding bioethanol at 0.08–0.10 g/g dry biomass. These values are around half as large as the benchmark for first-generation bioethanol. One of the problems with pressurised disc refining is that only a small amount of the cellulose appears to be hydrolysed to glucose, at least using the conditions employed in this work; strategies to improve cellulose decomposition could involve the use of additional enzymes (such as cellubiases), or even through enzymatic hydrolysis prior to pressure refining. This work has illustrated the potential utility of electrochemical methods for the on-line and real-time detection requirements of process analytical technology (PAT) suitable for Industry 4.0.

## CRediT authorship contribution statement

**Rhys A. Ward:** Validation, Formal analysis, Investigation, Data curation, Writing - original draft, Visualization. **Adam Charlton:** Conceptualization, Methodology, Formal analysis, Investigation, Resources, Visualization, Supervision, Project administration, Funding acquisition. **Kevin J. Welham:** Methodology, Formal analysis, Investigation. **Paul Baker:** Conceptualization, Formal analysis, Investigation, Resources, Project administration, Funding acquisition. **Sharif H. Zein:** Conceptualization, Resources, Project administration, Funding acquisition. **Jeremy Tomkinson:** Conceptualization, Resources, Project administration, Funding acquisition. **David I. Richards:** Conceptualization, Resources, Project administration, Funding acquisition. **Stephen M. Kelly:** Conceptualization, Methodology, Resources, Supervision, Project administration, Funding acquisition. **Nathan S. Lawrence:** Conceptualization, Methodology, Validation, Formal analysis, Resources, Supervision, Project administration, Funding acquisition. **Jay D. Wadhawan:** Conceptualization, Methodology, Validation, Formal analysis, Investigation, Resources, Data curation, Writing - original draft, Visualization, Supervision, Project administration, Funding acquisition.

## Declaration of Competing Interest

The authors declare that they have no known competing financial interests or personal relationships that could have appeared to influence the work reported in this paper.
