# Supplementary material for: Electrochemical quantification of d-glucose during the production of bioethanol from thermo-mechanically pre-treated wheat straw
Source: Electrochem commun. 2021 Mar;124:106942. doi: 10.1016/j.elecom.2021.106942 (PMC7976448; doi:10.1016/j.elecom.2021.106942)
Supplement: Supplementary Data 1 [file mmc1.pdf]

# Electrochemical Quantification of D-Glucose during the Production of Bioethanol from Thermo-Mechanically Pre-treated Wheat Straw

Rhys A. Ward,[1,2] Adam Charlton,[3*] Kevin J. Welham,[4] Paul Baker,[3]
Sharif H. Zein,[1,2] Jeremy Tomkinson,[5] David I. Richards,[2]
Stephen M. Kelly,[2,4] Nathan S. Lawrence,[1,2] Jay D. Wadhawan[1,2*]

[1]*Department of Chemical Engineering, The University of Hull, Cottingham Road, Kingston-upon-Hull HU6 7RX, United Kingdom.*

[2]*Aura Innovation Centre, Bridgehead Business Park, Meadow Road, Hessle HU13 0GD, United Kingdom.*

[3]*BioComposites Centre, Bangor University, Alun Roberts Building, Bangor, Gwynedd LL57 2UW, Wales, United Kingdom.*

[4]*Department of Chemistry and Biochemistry, The University of Hull, Cottingham Road, Kingston-upon-Hull HU6 7RX, United Kingdom.*

[5]*National Non-Foods Crops Centre (NNFCC), Ltd., Biocentre, York Science Park, Innovation Way, York YO10 5DG, United Kingdom.*

[*]Corresponding authors.
E.mail: adam.charlton@bangor.ac.uk (AJC); j.wadhawan@hull.ac.uk (JDW)

## Supplementary Information (SI)

**SI1:** *Experimental Section* S2

**SI2:** *Quantification of D-Glucose* S7

**SI3:** *Scale Dependence of D-Glucose Productivity* S11

**SI4:** *Detection and Determination of Ethanol* S13

**SI5:** *SI References* S16

## SI1: Experimental

*Chemical and Biochemical Reagents*

Wheat straw (with expected composition of 30-45% cellulose, 20-25% hemicellulose and 15-20% lignin [S1]) was harvested in the East of England. Cellulase used for the enzymatic hydrolysis of the pressure-refined wheat straw was obtained from the National Centre for Biotechnology Education (NCBE) at The University of Reading in the form of Celluclast® 1.5 L (Novozymes A/S, Bagsværd, Denmark) – an aqueous enzyme preparation (aep) produced by a selected strain of the fungus *Trichoderma reesei*, containing 10-20 wt.% cellulose (aep). Slaked lime was a generous gift from Singleton Birch, Ltd. All other reagents were obtained from Sigma-Aldrich in their highest commercially available grade, and used without further purification. These included *Saccharomyces cerevisæ* Type II yeast, glucose-1-oxidase from *Aspergillus niger* (EC 1.1.3.4), Type X-S, lyophilized powder, of activity either between 0.1 – 0.25 MU/g solid, or 17.3 kU/g solid, both without added oxygen, where 1 U oxidises 1.0 μmol of β-D-glucose to D-gluconic acid and water per minute at 25°C. Water, with a resistivity of not less than 18 MΩ cm, was taken from an Elgastat system (Vivendi, Bucks., UK). Nitrogen gas, used for purging electrochemical solutions, was supplied by Energas (Air Liquide). All experiments were undertaken at ambient temperature (21 ± 3°C), unless stated otherwise.

*Preparation and Pre-treatment of Wheat Straw*

The pre-treatment protocols of the wheat straw used in this work have been reported previously[S2] and summarised here. The wheat straw was transported to the refiner at the BioComposites Centre's Biorefining Technology Transfer Centre (Anglesey, Wales), where it was processed using an Andritz pressure disc refiner. In order to prevent "bridging" of material in the feed hopper (*vide infra*), and prevent issues with the longer fibres wrapping around and clogging the feedscrew in the modular screw device (MSD), all of the straw was pre-processed using a Pierret N40 forage chopper set to a 0.5 in (12.7 mm) cut length.

Wheat straw samples were either pre-treated using atmospheric disc refining – a standard process used in the paper and pulp industry to increase the surface area of the substrate and disrupt fibre bundles (shives), or through treatment at higher pressures. The atmospheric disc refiner used (Sprout Waldron 12" model) comprised three components: a rotating disc plate connected to the motor, a stationary disc plate, and housing. The disc plates were enclosed within the housing and had a series of surface groves and bars, which when brought together during the refining process resulted in a physical change to the fibre structure. The forage chopped wheat straw fibre was suspended in hot water at 55°C for 1 h, prior to pre-treatment, and the subsequently refined fibre was drained using a fine wire mesh screen (149 μm) to remove excess water, and then de-watered using a Vincent CP4 screw press, after which it was bagged and either sent for analysis, or frozen (-10°C).

The continuous pressurised disc refining equipment has previously described [S2]. The process illustrated in Figure 1 of the main paper was employed. Briefly, the chopped straw

was fed through a hopper, into a screw feeder or modular screw device (MSD), where a biomass "plug" was formed and transported into a heated cooker screw and, subsequently, into a 60 L digester. The steam-treated biomass was then fed into the pressure refining zone, consisting of two 12 inch (diameter) Andritz refining plates (either D2-516 – typical of those used for the preparation of medium density fibreboard, or D2-503 – patterned bi-directionally with a bar angle of 0° and designed with more emphasis on grinding), one of which was fixed in position while the other rotated at 2500 rpm using a specified plate gap. The fibre was ejected from the refiner and into a collection bin through a cyclone, where it was bagged, weighed and either sent for analysis, or frozen (-10°C ). Table 1 of the main paper summarises the pre-treatment conditions for the wheat straw samples employed within this work.

*Enzymatic Hydrolysis of Thermo-Mechanically-Refined Wheat Straw*

Frozen fibres were defrosted and weighed at ambient temperature prior to hydrolysis with small scale (25 mL), triplicate experiments, undertaken to assess the effect of refiner pressure on D-glucose yields. Larger scale (0.5 – 5.0 L) studies investigated the scalability of the process were undertaken only using wheat straw refined at 10 bar. All experiments were undertaken using the same ratios of straw-to-water and Celluclast® 1.5 L-to-water ratios. A slurry of 0.02 g $mL^{-1}$ of wheat straw in 10 mM aqueous phosphate citrate buffer solution (comprising 5 mM $KH_2PO_4$ and 5 mM citric acid) at pH 5 - 6 was prepared and placed into a round-bottomed flask containing 2.1% vol./vol. Celluclast® 1.5 L enzyme. For the small scale experiments (25 mL), the volume of the aep solution was 525 μL, corresponding to 10 filter paper units (FPU) [S2,S3]. The weighed flasks were fitted with a condenser to minimise evaporative losses, stirred (260 rpm) and heated in a water bath (50 ± 5 °C) for 72 h. For the larger scale studies, a 1.0 L flask was used for 0.5 L solutions; a 2.0 L flask was used for 1.0 L solutions; and a 2.0 L flask was used for 2.0 L solutions. Due to the quantity of refined straw (100 g) used for the 5.0 L solution experiments, these were conducted in a 6.0 L glass beaker, with mechanical stirring (120 rpm, using a four-blade, 6 cm diameter, 45° pitch-bladed turbine impeller, suitable for axial flow bulk fluid mixing). The vessel was covered with aluminium foil to reduce evaporative losses.

After 72 h, the flasks were reweighed, a 1.5 mL aliquot taken and passed through a 0.22 μm filter. The sample was centrifuged (13000 rpm at 20°C, 5 min), and the resulting supernatant was either analysed immediately for D-glucose content, or frozen and stored at -20°C, for analysis at a later date. The remaining slurry was immediately used in the fermentation step.

*Analytical Quantification of D-Glucose in Hydrolysate*

An electrochemical procedure using cyclic voltammetry was used for the quantification of D-glucose in the hydrolysate, based on the protocols developed to monitor blood glucose levels [S4]. The physicochemical rationale underpinning this measurement is discussed in SI2. The electrochemical system comprised a traditional three-electrode cell (5.0 or 50.0 mL volume),

employing a 3 mm diameter glassy carbon working electrode (Bioanalytical Systems), a graphite counter electrode and a saturated calomel reference electrode (SCE, Radiometer), controlled by a μAutolab Type III potentiostat (Eco Chemie). Experiments were undertaken within a small (5 mL) phosphate buffer solution at pH 6.74, comprising 50 mM $KH_2PO_4$ and 50 mM $Na_2HPO_4$ containing 0.25-0.26 mM ferrocenemethanol and either 1.0 mg/mL (for kinetic measurements) or 0.1 mg/mL (for analytical measurements) glucose 1-oxidase.

Cyclic voltammograms of ferrocenemethanol were recorded at a scan rate of 0.1 V/s, in both the absence and presence of D-glucose. The solutions were oxygen-purged through degassing with a stream of nitrogen for at least ten minutes, and maintaining a blanket of nitrogen over the solution during experimentation. Calibration curves were generated using standard method additions and monitoring the oxidative peak current increase with the addition of aliquots (either 10 or 100 μL) of a 1.0 M D-glucose solution that had been prepared 24 h in advance (to allow for the mutarotation equilibrium to be established). Prior to recording each voltammogram, the working electrode was washed and polished with 0.3 μm alumina slurry on a wetted, napped polishing cloth rotating at 150 rpm (MECAPOL P230, Presi, France), before being rinsed with water, dried and used.

The analysis of the hydrolysates followed a similar procedure to that for the calibration, except that the additions of mutarotated 1.0 M D-glucose solution were replaced with 100 μL aliquots of the centrifuged and filtered hydrolysates. These samples were considered to be at anomeric equilibrium; additions were performed in duplicate.

*Fermentation of Hydrolysate*

The hydrolysate was filtered under vacuum (using 110 mm diameter Satorius grade 292 Büchner filtration discs) to remove undigested straw debris. The filter was changed to a plug of glass wire wool for experiments at the largest volumes (5.0 L). Fermentation of the filtrate was undertaken using 0.03 g/mL of dry yeast (*S. cerevisæ, type II*) which had been reconstituted in 150 mL of distilled water for at least 60 min prior to use [S5]. Fermentation experiments were conducted using a conical flask with side arm, fitted with an air lock formed by keeping a rubber bung in the neck of the flask and feeding the side arm to a Dreschel bottle containing water saturated with slaked lime. This also enabled any carbon dioxide generated through fermentation to be readily neutralised. This fermentation stage was undertaken through incubation in a water bath (36$^{o}$C, 48 h), with stirring (600 rpm). For the larger scale experiments, fermentation was undertaken using a 5.0 L round-bottomed flask with the neck sealed as before with agitation.

After 48 h, the final volume of the fermentation broth was recorded, and a 1.5 mL sample of the fermentate was taken, filtered through a 0.22 μm filter, and either assessed immediately

for ethanol content, or frozen and stored at -20°C, for quantitative analysis at a later date by gas chromatography-mass spectrometry.

*Semi-Qualitative Determination of Ethanol in Fermentate*

In order to identify, qualitatively, the presence of ethanol in the fermentate, a visual assay was employed, wherein a cloudy, ternary mixture of oleic acid, water and ethanol transforms into a clear and colourless microemulsion (*q.v.* Figure S1) upon the addition of a critical amount of ethanol. This enables a ratio to be determined between the concentration of oleic acid and ethanol at the cross-over point. This protocol was used for the qualitative assessment of ethanol within the fermentate [S6], with the physicochemical rationale underpinning this method discussed in SI4. Note that it assumes there is a constant amount of oleic acid in the samples [S7]. Equal volumes (300 µL) of oleic acid and an aqueous solution (pure water, or aqueous buffer, or aqueous buffer containing Celluclast® 1.5 L) containing a known amount of ethanol were vigorously agitated using a Labnet vortex mixer, to afford a cloudy, turbid emulsion. 100 µL aliquots of absolute ethanol were gradually added to this emulsion and agitated vigorously using the vortex mixer, to observe the range corresponding to the transition between the cloudy emulsion and the point of microemulsification. Once known, a more accurate assessment was made, in a separate flask with a second sample, using 10 µL additions of absolute ethanol to a fresh equivolume mix, starting from 50 µL below the observed transition. For analysing the fermentate samples, the 300 µL aqueous phase corresponded to the fermentation broth; measurements were undertaken in triplicate.

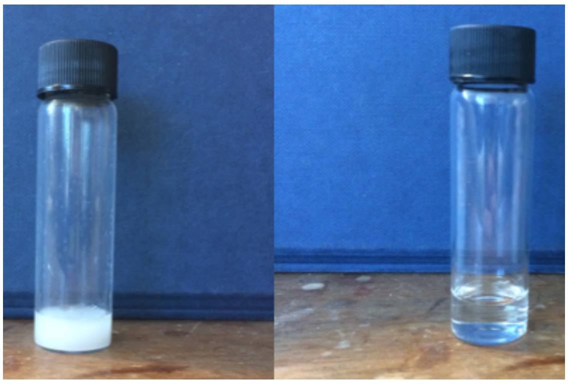

***Fig. S1:*** *Addition of sufficient ethanol to an unstable, milky white emulsion formed from equivolumes of oleic acid and water (left) results in the formation of a stable, clear and colourless microemulsion (right).*

*Quantitative Determination of Ethanol in Fermentate*

Quantitative analysis of fermentation samples was undertaken using gas chromatography-mass spectrometry (GC-MS), with an internal standard, using a conventional method [S8]. 1.5 mL samples were made-up using 0.5 vol.% *iso*-propyl alcohol as the internal standard, with the remainder being either an aqueous-ethanol mixture (for calibration) or the fermentation broth. A Perkin Elmer Turbomass quadrupole mass spectrometer with autosystem XL GC was employed. Two types of wall coated open tubular (WCOT) columns were used: either a Thames Restek RXI 5MS column, or a Thames Restek stabilowax column.

(30 m length; 0.2 mm internal diameter; stationary phase of thickness 0.25 μm). The mobile phase was helium (0.5 mL/min), with an injection volume of 0.5 μL (split ratio of 250 : 1). Injection syringes were cleaned with methanol prior to sampling. The mass spectrometer was set to scan in the range $15 \leq m/z \leq 100$ every 0.5 s. No solvent cut time was employed; the measurement was timed to start with the injection.

## SI2: Quantification of D-Glucose

Enzymatic hydrolysis of lignocellulosic material liberates a number of hexose (D-glucose, D-mannose, D-galactose and D-fructose) and pentose (D-xylose and D-arabinose) monosaccharides [S1]. Accordingly, as indicated in the main paper, an index of the "fermentability" of a particular hydrolysate broth is the D-glucose level, since all yeasts have hexakinases which metabolise this molecule [S9]. However, in aqueous solution, D-glucose exists in an equilibrium mixture of a variety of structures, but principally within two anomeric, pyranose forms: α-D-glucose (36%) and β-D-glucose (63%), *q.v.* Figure S2. Yeast cells typically metabolise the β-anomer faster than the α-anomer [S10], consistent with the anomeric difference in the rate of oxidation [S11].

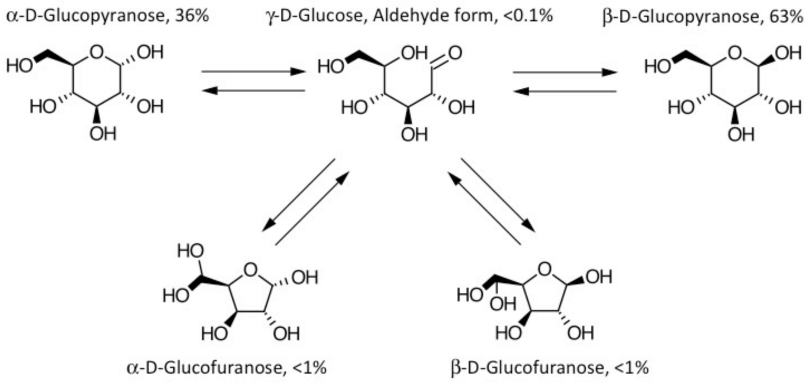


***Fig. S2:*** *Structural equilibria pertaining to D-glucose in aqueous solution. The quantities indicated correspond to the amounts at 20 °C. Although,* stricto sensu*, mutarotation refers to the change in optical rotation of different anomers, these terms to refer to the development of D-gluocose in its equilibrium mixture of forms.*

Numerous literature methods have been reported for the quantification of the liberated monosaccharides after enzymatic hydrolysis [S1,S2,S12]. However, the majority of such studies exploit either colorimetric reducing sugar analysis (which cannot discriminate between different monosaccharides) or liquid chromatography with detection through changes in refractive index [S13]. These methods have limitations because they offer little discrimination between D-glucose and other monosaccharides. It follows that methods employed for accurate blood glucose measurement [S4,S11,S14], through the use of electrochemical enzymatic assays and adapted for this study, would provide and accurate and reliable quantitative method.

Several electrochemical enzymatic methods have been proposed for measuring D-glucose, utilising either glucose dehydrogenases or glucose oxidases [S15]. These are both specific for the β-anomer of glucose (note that glucose-2-oxidase is not specific to the β-anomer) [S16].

However, whilst the dehydrogenases have a higher turnover, they typically either require a soluble co-factor, or present stability issues precluding their use [S17]. Accordingly, glucose-1-oxidase (GOx, EC 1.1.3.4, with molecular mass reported in the range 151-186 kDa, with most measurements typically 155 ± 5 kDa [S16]) is the preferred oxidoreductase to monitor β-D-glucose, through its transformation into glucono-δ-lactone (which subsequently hydrolyses to D-gluconic acid), concomitantly reducing molecular oxygen to hydrogen peroxide. Note though, as indicated in the main paper, this enzyme also holds activity for other sugars derived from hydrolysed hemicelluloses (*viz.* D-xylose, D-mannose, D-galactose, with negative activity – inhibition – for D-arabinose), but these are with two orders of magnitude less than the activity towards D-glucose).

Commercial analysers used for D-glucose concentration analysis in fermentation samples [S14], often exploit the amperometric monitoring of hydrogen peroxide, as developed by Clark and Lyons [S17]. However, this is flawed since hydrogen peroxide oxidation required the use of high potentials, (thereby encouraging interferences with the measurement), produces oxygen as the electrode reaction (which may feedback and change the rate of D-glucose consumption by GOx), and is highly pH sensitive, with the pH being altered close to the electrode surface through proton liberation [S11]. Accordingly, a more favoured route is through the use of soluble one-electron homogeneous mediators, such as ferricenium ions, switching the flavin adenine dinucleotide (FAD) moieties within the active site of GOx between redox states, as illustrated in Figure S3. This system is attractive, since the homogeneous redox catalysis means that the oxidation can be achieved at much lower potentials than hydrogen peroxide oxidation (typically lower than 0.4 V, since the formal potential for ferrocenemethanol is 0.19 V *vs.* SCE), thereby reducing the extent by which interferences affect the measured signal.

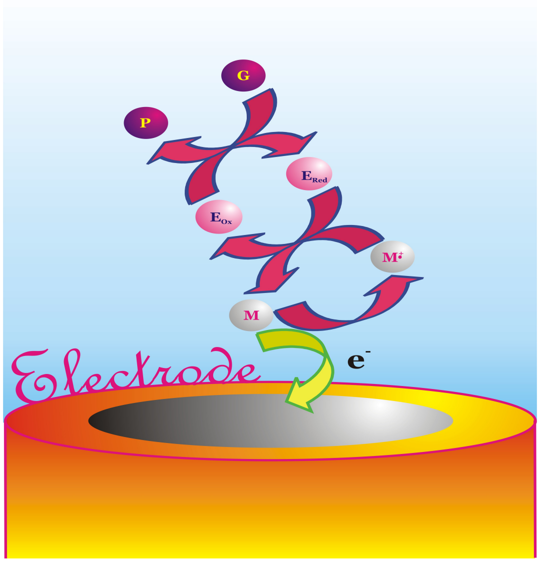


***Fig. S3:*** *Homogeneous redox-catalysed oxidation scheme for amperometric D-glucose monitoring. In this scheme, G refers to β-D-glucose, P is glucono-δ-lactone, $E_{Ox}$ and $E_{red}$ refer to the oxidised (FAD) and reduced ($FADH_2$) forms of GOx, and M and $M^{•+}$ refer to ferrocenemethanol and ferriceniummethanol, respectively.*

Figure S4a illustrates typical cyclic voltammograms (recorded at 0.1 V/s) corresponding to the additions of β-D-glucose to a buffered solution of ferrocenemethanol containing GOx at pH 6.74. It is clear that the waveshape alters through transition between the classical peak-shaped response and a sigmoidal shape with increase in β-D-glucose. The observed maximum current in the presence of β-D-glucose ($i_p$), expressed relative to that in its absence ($i_p^0$) is the current turnover number, T/N:

$$T / N = \frac{i_p}{i_p^0} \tag{S1}$$

and is depicted in Figure S4b as a function of the concentration of β-D-glucose present in the solution. Note that these curves were recorded with two particular additional experimental requirements: first, owing to chloride-induced ferricenium decomposition [S18], and the inhibition of GOx by halide ions [S16], the phosphate buffer solutions rigorously excluded the chloride salts; second, thorough oxygen-purging of the solution was required since $O_2$ is the natural oxidant for GOx (Michaelis constant of 0.2 mM [S15]).

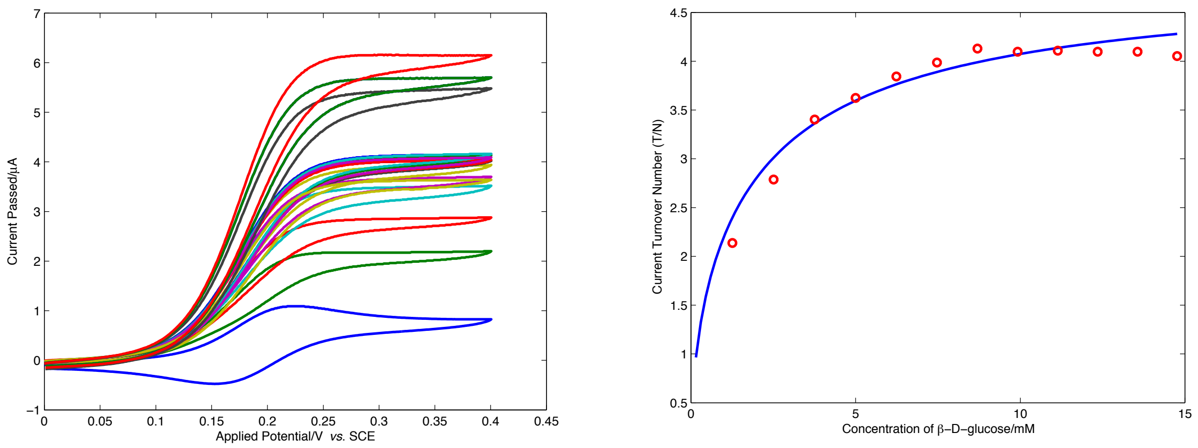


***Fig. S4:*** *Cyclic voltammograms (0.1 V s⁻¹) at a 3 mm diameter glassy carbon electrode of 0.25 mM ferrocenemethanol in 0.1 M aqueous phosphate buffer at pH 6.74 and 27°C containing 1.0 mg mL⁻¹ GOx (a, left), with and without 100 µL standard additions of a 1.0 M aqueous glucose solution (mutarotated). Panel (b, right) illustrates the analysis of the peak or plateau in the voltammograms through the current turnover expression given by equation (1), red circles; the blue line is the theoretical fit with equation (S2), with a coefficient of determination, $R^2$ = 0.9459, defined as* $R^2 = 1 - \frac{\text{residual sum of squares}}{\text{total sum of squares}}$. *Note that the analysis assumes the occurrence of 63% β-anomer.*

Savéant *et al.* examined the mechanism for this modified Michealis-Menton reaction scheme, and assumed that all forms (free and bound to β-D-glucose) of the redox-active site in GOx are at steady-state and that the enzyme is immobile compared with both redox forms of ferrocenemethanol [S19]. This approximation is valid since the mediator has a diffusion coefficient that is an order of magnitude larger than that of the enzyme [S16], *c.f.* 7 x $10^{-6}$ *vs.* 4 x $10^{-7}$ $cm^2$ $s^{-1}$. Therefore, provided the enzyme activity is sufficiently low, so that there is little β-D-glucose depletion within the reaction layer at the electrode surface (corresponding

to the “no substrate consumption” area of the kinetic zone diagram), the increase in peak current for cyclic voltammetry in the presence of D-glucose compared with its absence, is readily derived and is given by the equation,

$$T/N = \frac{\sqrt{2}}{0.4463}\sqrt{\frac{k_3 c_E^0}{v}}\sqrt{\frac{RT}{F}}\sqrt{\frac{2k_2}{k_3 c_P^0\left(1+\frac{K_M}{c_G^0}\right)}\left\{1-\frac{k_2}{k_3 c_P^0\left(1+\frac{K_M}{c_G^0}\right)}\ln\left[1+\frac{k_3 c_P^0}{k_2}\left(1+\frac{K_M}{c_G^0}\right)\right]\right\}} \tag{S2}$$

in which $c_E^o$, $c_P^o$ and $c_G^o$ respectively refer to the concentrations of the total enzyme (GOx), ferrocenemethanol and β-D-glucose; R is the molar gas constant, F is Faraday’s constant and T is the absolute temperature; $k_2$ is the rate constant for the breakdown of the enzyme-glucose complex within the Michealis-Menton scheme, for which the Michealis constant is $K_M$, and $k_3$ is the bimolecular rate constant for the recycling of the $FADH_2$ moiety within the active site of GOx to FAD. The factor of two present in equation (S2) recognising the fact that the FAD/$FADH_2$ couple involves two electrons, whilst the mediator is a one-electron oxidant. Taylor expansion of the Naperian logarithmic term in equation (S2) affords the expression:

$$T/N = 3.1688\sqrt{\frac{RTc_E^0 k_3}{Fv}} \tag{S3}$$

which is valid at high glucose concentrations, and from which $k_3$ is determined to be in agreement with literature (2.1 x $10^5$ $M^{-1}$ $s^{-1}$ *vs.* 9.0 x $10^3 \leq k_3/$ $M^{-1}$ $s^{-1} \leq$ 3.1 x $10^7$) [S20]. Hence, using equation (S2), as indicated in Figure S4b, the experimental data conform to $k_2$ = 20 $s^{-1}$ $K_M$ = 14 mM, which is expected ($95 \leq k_2/s^{-1} \leq 770$; $1.0 \leq K_M/mM \leq 100$) [S20], testifying to the correct operation of the electrochemical method.

As already outlined, D-Glucose is not the only sugar that is liberated through enzymatic hydrolysis of lignocellulosic biomass: celluloses and hemicelluloses are first hydrolysed to soluble oligomers, and subsequently further hydrolysed to afford a range of monosaccharaides including D-glucose, D-xylose, D-mannose and D-galactose [S1]. The hydrolytic procedure employed in this study involved the use of only one enzyme, Celluclast 1.5 L, which is known to contain a diverse spectrum of cellulolytic enzyme activities, including cellobiohydrolases and endo-1,4-β-glucanases [S21,S22], and is able to breakdown cellulose into D-glucose, cellobiose and longer glucose polymers, with reported yields of 52% for wheat straw [S22]. It cannot breakdown cellobiose, so it is important to realise that the D-glucose yield might have been increased through the additional use of β-glucosidase; in fact, the enzyme manufacturer recommends the use of a particular cellubiase, Novozym 188; further details may be obtained from the supplier electronically: ncbe@reading.ac.uk.

## SI3: Scale Dependence of D-Glucose Productivity

As outlined in the main paper, there the D-glucose productivity is approximately constant over the reported scale, except at the largest of the scales used. At that scale, the mixing regime used was slightly different to the smaller scale experiments, and this impacts through altering the mass transfer coefficient, as outlined below.

The mass transfer coefficient from particles ($k_{sL}$) in turbulent flow is thought to obey the relationship [S23],

$$k_{sL} = 0.693\left(\frac{D^2}{x}\right)^{1/3}\left(\varepsilon\frac{\rho}{\mu}\right)^{1/6} \qquad \text{(S4)}$$

where x is the (monodispersed) straw particle diameter (taken to be between 50 – 1000 μm), D is the diffusion coefficient of material from the particle, ρ is the fluid density (taken as 1000 kg/m$^3$ for water), μ is the dynamic viscosity of the fluid (taken as that of water, 1.0 cP, equivalent to $10^{-3}$ N s/m$^2$), and ε is the specific dissipated power on mixing (in W/kg) – the amount of mechanical energy converted to heat per unit time per unit solution mass. Equation (S4) is derived from the expression for the Nusselt number, Nu, equation (7.1) given in reference [S23] as $Nu = 0.55\left(\frac{a^2\varepsilon^{1/2}}{D\nu^{1/2}}\right)^{1/3}$ where a is the particle radius, ν is the kinematic viscosity of the bathing fluid, and the other symbols are defined in equation (S4). The derivation of equation (S4) starts by equating the Nusselt number to the Sherwood number, $Sh = \frac{k_{sL}\left(x/2\right)}{D}$, recognising that $a = \frac{x}{2}$. Both Folger and LeBlanc [S24] and Storck and Lapicque [S25] have illustrated how the mass transfer model described by equation (S4) can be adapted to account for polydisperse solids.

Although the kinematic viscosity (μ/ρ) will change slightly as the reaction proceeds, considering this and the particle size constant, all other things being approximately equal, given that the specific dissipated power varies as [S26,27],

$$\varepsilon \propto \frac{D^5}{D_T^2} \qquad \text{(S5)}$$

in which D is the impeller diameter, and $D_T$ is the tank size, the mass transfer coefficient for sugar hydrolysis then varies as the ratio:

$$k_{sL} \propto \frac{D^{5/6}}{D_T^{1/3}} \qquad \text{(S6)}$$

This ratio was not kept constant during our scaled experiments, so that the data in Figure 3 (panels b and c) of the main paper also reflect the different changes in mass transport; indeed, the Reynolds number (Re) for pitch-bladed turbine impellers is given by equation (S7):

$$\mathrm{Re} = \frac{D^2 N \rho}{\mu} \tag{S7}$$

where D is the impeller diameter (0.06 m), N is the agitator speed (120 rpm, equivalent to 2 rps) [S27]. For the small impeller employed in this work for the 5 L scale experiments, the Reynolds number is estimated as being 7200. This number is smaller than the 10000 that characterises turbulent flow and rigorous vertical mixing within the hydrolysis cell, but larger than 10 (the upper limit of laminar flow). Accordingly, this transient mixing regime is considered to exhibit turbulent flow at the impeller, but laminar flow in the remote parts of the hydrolysis vessel [S27]. This reduction in D-glucose level with non-turbulent flow reaffirms the results obtained by Battista and co-workers [S14], who observed that anchor and double helical impellers liberate over two-and-a-half times more D-glucose than pitch-bladed turbine impellers. However, better mixing is not without a costly economic drawback – the electrical power consumed by an agitator is proportional to the product $D^{5+2b+c}N^{3+b+2c}$ where b and c are indices to the Reynolds and Froude numbers, respectively, and are dependent on the agitator type, and the vessel-agitator dimensions and geometry [S28].

## SI4: Detection and Determination of Ethanol

In fermentation, hexoses are converted to ethanol through micro-organism metabolism:

$$C_6H_{12}O_6\left(aq\right)\xrightarrow{S.cerevisiae}2C_2H_5OH\left(aq\right)+2CO_2\left(g\right) \qquad \text{(S8)}$$

The yeast employed in this work (*Saccharomyces cerevisæ*) is known to consume both monosaccharides and disaccharides [S29], not just D-glucose as suggested by equation (S8). In considering this reaction for the lower levels of D-glucose generated by small laboratory-scale reactors, between 0.1-0.2 vol.% ethanol is expected, assuming only D-glucose is fermented at the stoichiometry in equation (S8), with the lower levels anticipated for the AR samples. Accordingly, qualitative comparative experiments (assuming there is an equal amount of oleic acid in all of the samples [S7]) were undertaken using a microemulsion method. The results are reported in Figure S5.

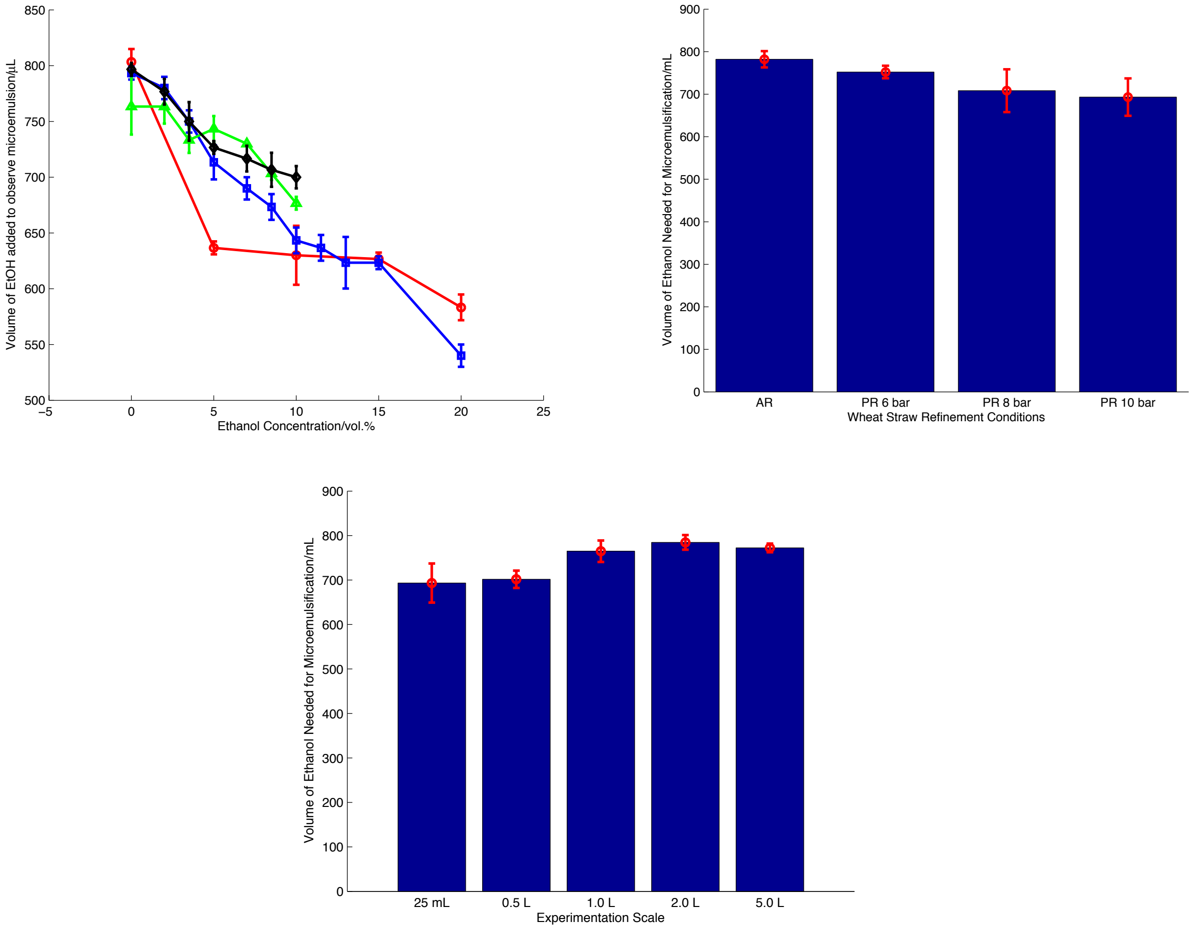


***Fig. S5:*** *(a, top left) Variation of the volume of absolute ethanol required to observed microemulsification through addition to a mixture containing 300 µL oleic acid and 300 µL of water (red circles), phosphate citrate buffer (blue squares and green triangles) or phosphate citrate buffer containing 2.1 vol.% Celluclast® 1.5 L (black diamonds) that already contained a known amount of ethanol. Experimental temperatures were 24 °C (red circles and blue squares), 19 °C (green triangles) and 18 °C (black diamonds). In each case, the error bars represent one standard deviation; a line is drawn through the experimental data to guide the eye. (b, top right) The amount of ethanol needed to be added to a mixture of 300 µL of oleic acid and 300 µL of broth resulting from 25 mL hydrolysis of control (AR) or pressure refined (PR) wheat straw followed but its fermentation: n=6 (AR), n=6 (PR6 bar), n=18 (PR8 bar), n=27 (PR10 bar). Experiments were undertaken at 20 ± 2 °C. Error bars represent one standard deviation. (c, bottom) The variation in the amount of ethanol required to take a mixture of 300 µL of oleic acid and 300 µL of broth obtained after hydrolysis of 10 bar pressure refined wheat straw undertaken at different experimental scales, followed by its fermentation: n=27 (25 mL), n=6 (0.5 L), n=21 (1.0 L), n=15 (2.0 L), n=6 (5.0 L). Experiments were conducted at 20 ± 2 °C. Error bars represent one standard deviation. In panels (b) and (c), n refers to the sample size.*

The microemulsion technique used in this work was adapted from that developed by Kubota *et al.* [S6]. It relies on the fact that water and ethanol are completely miscible, as are ethanol and oleic acid, whilst water and oleic acid form unstable emulsions [S6]. Thus, the ternary phase diagram of water-ethanol-oleic acid comprises two main regions – a two phase region and a single phase region. The latter is a thermodynamically stable microemulsion, characterised by a clear and colourless single phase (*q.v.* Figure S1). It thus follows that a water and oleic acid emulsion containing small amounts of ethanol will exhibit two-phase characteristics, until a minimum volume of ethanol (considered as an amphiphile) is added to empower nanodroplet formation by reducing the Gibbs energy for the dispersion. This is manifested as a cloudy-to-transparent transition, with the analytical signal being the threshold volume of ethanol added, *q.v.* Figure S5a.

It is clear from Figure S5a that the higher the ethanol content within the aqueous phase, the lower the amount of ethanol is required to elicit microemulsification. However, this method, whilst facile in implementation, can only provide a qualitative assessment for the amount of ethanol present with the fermentation broth, since, as illustrated in Figure S5a, the microemulsification transition point is extremely sensitive to the presence of inorganic salts and organic entities (including proteins), in addition to temperature.

Thus, considering the series of experiments undertaken at constant volume hydrolysis, with variation of the type of biomass refinement (Figure S5b), it is clear that the samples treated at higher pressures (8 bar or 10 bar) afforded higher ethanol yields. This is in agreement with the conclusions from the D-glucose analysis, where the control experiments afforded the smallest amounts of D-glucose. However, given that the D-glucose level is approximately constant for all PR samples, the worse performance by the PR6 bar samples reaffirms the decision to test for scalability using PR10 bar samples, and is in agreement with our earlier results that a greater amount of total monosaccharides is released from fibres refined at higher pressure [S2]. Accordingly, consideration of the data reported in Figure S5c indicates that fermentation efficiency worsens as the experimental scale increases, although this experiment does not take into consideration any naturally occurring oleic acid derived from the wheat straw [S7].

In order to quantify the amount of ethanol produced within the larger scale experiments, a GC-MS method was employed. Gas chromatograms of samples containing an internal standard (*iso*-propyl alcohol) were recorded for a set of standard samples, to afford calibration graphs similar to that depicted in Figure S6a, with peak areas estimated through fitting the individual peaks to a Gaussian curve followed by integration. Figure S6b shows that ethanol is produced from the fermentation process, albeit at 0.22 ± 0.12 vol.% (range 0.03 - 0.47 vol.%), giving rise to a space-time yield of 37.1 ± 22.9 mg $L^{-1}$ $h^{-1}$ for ethanol production (*q.v.* Figure S6c).

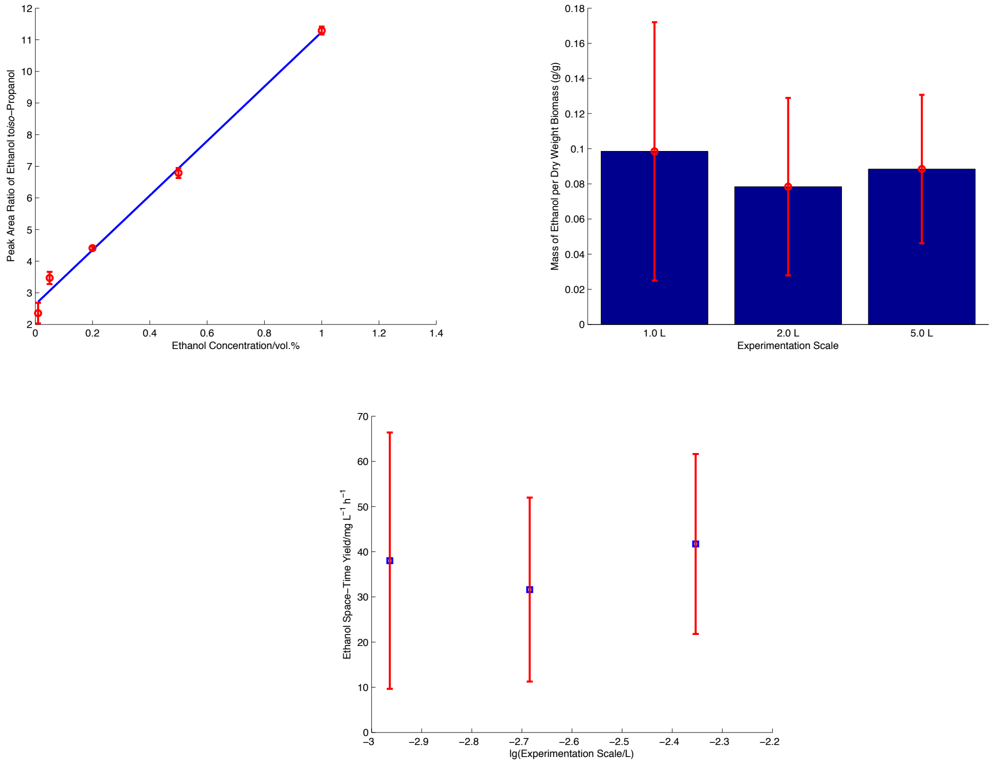


***Fig. S6:*** (a, top left) GC-MS calibration curve using 0.5 vol.% *iso*-propyl alcohol as an internal standard, analysed in triplicate. Experimental points are plotted as red circles; the blue line of best-fit is of the form $\text{Area Ratio} = 8.6121 \times \text{Ethanol Concentration} + 2.6320$ and holds a coefficient of determination of $R^2 = 0.9936$. (b, top right) Bioethanol mass yield normalised by the mass of PR10 wheat straw used (dry weight). (c, bottom) Variation of the space-time yield for bioethanol with experimental scale. In (b) and (c), the number of samples studied (n) were 4 (1.0 L), 5 (2.0 L) and 3 (5.0 L), each analysed in triplicate. The red error bars indicate one standard deviation.

## SI5: SI References


S1. (a) F. H. Isikgor, C. R. Becer, *Polym. Chem.*, 6 (2015) 4497-559; (b) P. Maki-Arvela, T. Salmi, B. Holmbom, S. Willfor, D. Y. Murzin, *Chem. Rev.*, 111 (2011) 5638-66; (c) J. B. Binder, R. T. Raines, *Proc. Nat. Acad. Sci.*, 107 (2010) 4516-21.

S2. C. Skinner, P. Baker, J. Tomkinson, D. Richards, A. Charlton, *Biores. Technol.*, 304 (2020) 122976.

S3. B. Adney, J. Baker, *Measurement of Cellulase Activities: Laboratory Analytical Procedure*, NREL Technical Report NREL/TP-510-42628, National Renewable Energy Laboratory, Golden, Colarado, 2008.

S4. S. B. Bankar, M. V. Bule, R. S. Singhal, L. Ananthanarayan, *Biotechnol. Adv.*, 27 (2009) 489-501.

S5. C. Bibby, *Simple Experiments in Biology*, William Heinemann, London, 1943, p.93 *f.f.*

S6. (a) R. S. Lima, L. Y. Shiroma, A. V. N. C. Teixeira, J. R. de Toledo, B. C. do Couto, R. M. de Carvalho, E. Carrilho, L. T. Kubota, A. L. Gobbi, *Anal. Chem.*, 86 (2014) 9082-90; (b) G. F. Giordano, L. Y. Shiroma, A. L. Gobbi, L. T. Kubota, R. S. Lima, *Anal. Methods*, 7 (2015) 10061-66.

S7. J. C. del Río, P. Prinsen, A. Gutiérrez, *J. Agricult. Food Chem.*, 61 (2013) 1904-13.

S8. G. K. Buckee, A. P. Mundy, *J. Inst. Brew.*, 99 (1993) 381-4.

S9. J. Okunda, I. Miwa, K. Inagaki, *Enzyme*, 31 (1984) 176-80.

S10. F. Malaisse, Lagae, M. X. Giroix, A Senner, W. J. Malaisse, *Biol. Chem. Hoppe. Seyler.*, 31 (1986) 411-6.

S11. P. N. Bartlett, K. F. E. Pratt, *Biosens. Bioelectron.*, 8 (1993) 451-62.

S12. *SSF Experimental Protocols – Lignocellulosic Biomass Hydrolysis and Fermentation*, NREL Analytical Procedure, National Renewable Energy Laboratory, Golden, Colarado, USA, 2001.

S13. (a) For colorimetric methods, see, for example (b) and (c); (b) G. L. Miller, *Anal. Chem.*, 31 (1959), 426-8; (c) M. Dubois, K. A. Gilles, J. K. Hamilton, P. A. Rebers, F. Smith, *ibid.*, 28 (1956) 350-6; (d) for refractive index methods, see, for example (e); (e) H. Mikami, Y. Ishida, *Bunseki Kagaku.*, 32 (1983) E207-10.

S14. F. Battista, M. G. Almendros, R. Rousset, P.-A. Bouillon, *Renew. Energ.*, 131 (2019) 152-8.

S15. J. M. Montornes, M. S. Vreeke, I. Katakis, in P. N. Bartlett (ed.), *Bioelectrochemistry*, John Wiley, Chichester, 2008, p.199.

S16. R. Wilson, A. P. F. Turner, *Biosens. Bioelectron.*, 7 (1992) 165-85.

S17. L. Clark, C. Lyons, *Ann. New York Acad. Sci.*, 102 (1962) 29-45.

S18. K. J. Wright, Z. O. Oiaidha, D. P. Love, M. Aljohani, G. M. Greenway, J. D. Wadhawan, *New J. Chem.*, 42 (2018) 18641-8.

S19. C. Bourdillon, C. Demaille, J. Moiroux, J.-M. Savéant, *J. Am. Chem. Soc.*, 115 (1993) 2-10.

S20. P. N. Bartlett, C. S. Toh, E. J. Calvo, V. Flexer, in in P. N. Bartlett (ed.), *Bioelectrochemistry*, John Wiley, Chichester, 2008, p.267.

S21. A. C. Rodrigues, M. Ø. Haven, J. Lindedam, C. Felby, M. Gama, *Enzym. Microb. Technol.*, 79-80 (2015) 70-7.

S22. L. Rosgaard, S. Pedersen, J. Langston, D. Akerhielm, J. R. Cherry, A. S. Meyer, *Biotechnol. Prog.*, 23 (2007) 1270-6.

S23. G. K. Batchelor, *J. Fluid Mech.*, 98 (1980) 609-23.

S24. S. E. LeBlanc, H. S. Folger, *A. I. Ch. E. Journal*, 33 (1987) 54-63.

S25. F. Lapicque, A. Storck, *Ind. Eng. Chem. Res.*, 27 (1988) 451-7.

S26. F. A. Holland, F. S. Chapman, *Liquid Mixing and Processing in Stirred Tanks*, Reinhold Publishing Corporation, USA, 1966.

S27. R. K. Sinnott, *Coulson and Richardson's Chemical Engineering*, volume 6, 2nd edn., 1993.

S28. J. H. Rushton, E. W. Costich, H. J. Everett, *Chem. Eng. Prog.*, 46 (1950) 467-76.

S29. R. Lagunas, *FEMS Microbiol. Rev.*, 10 (1993) 229-42.
